# Higher whole-blood selenium is associated with improved immune responses in footrot-affected sheep

**DOI:** 10.1186/1297-9716-42-99

**Published:** 2011-09-06

**Authors:** Jean A Hall, Rachel L Sendek, Rachel M Chinn, D Paul Bailey, Katie N Thonstad, Yongqiang Wang, Neil E Forsberg, William R Vorachek, Bernadette V Stang, Robert J Van Saun, Gerd Bobe

**Affiliations:** 1 Department of Biomedical Sciences, College of Veterinary Medicine, Oregon State University, Corvallis, OR 97331-4802, USA; 2 Department of Animal Sciences, College of Agriculture, Oregon State University, Corvallis, OR 97331-4802, USA; 3 Department of Clinical Sciences, College of Veterinary Medicine, Oregon State University, Corvallis, OR 97331-4802, USA; 4 Department of Veterinary & Biomedical Sciences, College of Agricultural Sciences, Pennsylvania State University, University Park, PA 16802, USA; 5 Linus Pauling Institute, Oregon State University, Corvallis, OR 97331-6512, USA

## Abstract

We reported previously that sheep affected with footrot (FR) have lower whole-blood selenium (WB-Se) concentrations and that parenteral Se-supplementation in conjunction with routine control practices accelerates recovery from FR. The purpose of this follow-up study was to investigate the mechanisms by which Se facilitates recovery from FR. Sheep affected with FR (*n *= 38) were injected monthly for 15 months with either 5 mg Se (FR-Se) or saline (FR-Sal), whereas 19 healthy sheep received no treatment. Adaptive immune function was evaluated after 3 months of Se supplementation by immunizing all sheep with a novel protein, keyhole limpet hemocyanin (KLH). The antibody titer and delayed-type hypersensitivity (DTH) skin test to KLH were used to assess humoral immunity and cell-mediated immunity, respectively. Innate immunity was evaluated after 3 months of Se supplementation by measuring intradermal responses to histamine 30 min after injection compared to KLH and saline, and after 15 months of Se supplementation by isolating neutrophils and measuring their bacterial killing ability and relative abundance of mRNA for genes associated with neutrophil migration. Compared to healthy sheep, immune responses to a novel protein were suppressed in FR-affected sheep with smaller decreases in FR-affected sheep that received Se or had WB-Se concentrations above 250 ng/mL at the time of the immune assays. Neutrophil function was suppressed in FR-affected sheep, but was not changed by Se supplementation or WB-Se status. Sheep FR is associated with depressed immune responses to a novel protein, which may be partly restored by improving WB-Se status (> 250 ng/mL).

## Introduction

Footrot (FR) is a common, contagious bacterial disease of sheep that results in lameness and large production losses for the sheep industry. Footrot is caused by infection with the bacterium *Dichelobacter nodosus*, a gram-negative, anaerobic and fastidious bacterium, in association with other bacteria, particularly *Fusobacterium necrophorum *[1-3]. When the interdigital skin of the foot is damaged or wet for a prolonged period of time, it may become invaded by the ubiquitous soil and fecal bacterium *F. necrophorum. F. necrophorum *causes interdigital dermatitis and produces toxins that cause necrosis of the superficial layer of the skin allowing co-infection with other bacteria such as *D. nodosus*. *D. nodosus *contains surface fimbriae and stable extracellular proteases that allow it to colonize the interdigital epithelial tissue, digesting the living dermis, and feeding on collagen [4,5]. A foul smell is associated with the accumulation of grey pasty exudate between the dermis and epidermal horn, and ultimately separation of the hoof horn from the underlying dermis occurs [5]. Although a program that requires strict culling during the hot, dry summer months (non-transmission period) has proven successful in eliminating FR in flocks in Western Australia [6], this protocol is unfeasible in other countries with cool, wet climates and widespread prevalence of infected flocks [5]. In these situations, management programs to control rather than eliminate infection are more important. Strategies include parenteral antibiotic treatment, topical antibacterial sprays, trimming of horn hoof, vaccination, low stocking density, and genetic selection for sheep breeds less susceptible to FR [5,7].

The role of the immune system in the etiology of FR is not well understood. Adaptive immunity, including humoral and cell mediated immunity (CMI), likely play a role in protection against FR [8,9], yet infected or vaccinated sheep do not develop long-term immunity and may become re-infected over time [5,10,11]. T-cell antigen presentation may be biased between strains of *D. nodosus *because vaccination with a polyvalent serotype vaccine does not protect against all strains equally [8]. Heritability of resistance to FR may be related to a specific range of MHC II haplotype that is required to generate a sufficient immune response to *D. nodosus *[9].

We reported previously that FR-affected sheep have lower whole-blood selenium (WB-Se) concentrations and that parenteral Se-supplementation in conjunction with routine control practices accelerates recovery from FR [12]. As a follow-up, we examined in this study the mechanisms by which Se may facilitate recovery from FR. Selenium deficiency results in immunosuppression and inhibits resistance to bacterial and viral infections, neutrophil function, antibody production, proliferation of T and B lymphocytes in response to mitogens, and cytodestruction by T lymphocytes and NK cells [13]. It is unknown whether Se supplementation to Se-replete ewes can improve innate immunity of neutrophils (bacterial killing) or acquired immunity, including humoral and CMI, in FR-affected sheep. We hypothesized that Se functions as an immunonutrient, and enhances both arms of the immune response and, thereby, accelerates the recovery from FR. Because immune responsiveness in sheep with FR, and the ability of Se to alter immune responses to a foreign protein in sheep with FR are not well understood, we used a broad approach. We measured the antibody titer and performed a delayed-type hypersensitivity (DTH) skin test to a novel protein, keyhole limpet hemocyanin (KLH), to assess humoral and CMI, respectively, in healthy and FR-affected sheep. Innate immunity was evaluated by measuring an intradermal histamine response, and by isolating neutrophils and assessing bacterial killing ability and relative abundance of mRNA for genes associated with neutrophil migration.

## Materials and methods

### Animals and study design

The animals and study design have been described in detail previously [12], including the WB-Se assay and foot scores. In short, this was a placebo-controlled clinical trial of 15-months duration involving crossbred, primarily black-faced mature ewes (age: 2-6 y; body weight: 100-110 kg). A commercial sheep flock was chosen for the project based on a high incidence of FR and the owner's willingness to participate in a long-term clinical trial. At the beginning of the study, FR-affected ewes (*n *= 38; the duration of FR in the sheep was unknown) were alternately randomized into two treatment groups and at 1-month intervals injected subcutaneously with 5 mg of Se (FR-Se; sodium selenite containing 45.6% Se; MU-Se,^® ^Schering-Plough Animal Health Corp., Union, NJ) or with 1 mL of saline solution (FR-Sal) for the duration of the study. A control group of healthy sheep without FR (*n *= 19) was also identified at the beginning of the study; they received no injections. Ear tags and paint brands were used to identify the ewes. Sheep feet were examined, trimmed, and scored for FR at 0, 3, 6, 9, and 15 months using a scale of 0 (no evidence of FR), 1 (interdigital dermatitis, presence of heat, and characteristic FR odor), 2 (initial underruning of the hoof wall between the toes), 3 (underrunning of the sole), and 4 (extensive underrunning of the sole and lateral walls of the hoof). The score of the foot with the highest lesion score was used for the statistical analysis. The experimental protocol was reviewed and approved by the Oregon State University Animal Care and Use Committee.

All sheep were fed primarily on pasture, except for a short period around lambing time in early winter when they were brought into the barn and fed hay silage. Routine farm management practices were not altered. A custom-made mineral supplement (Sonka Custom Mineral Premix, Wilbur-Ellis Company, Clackamas, OR, USA) that contained Se was provided free choice to all ewes throughout the duration of the study. The mineral supplement contained 5.0% calcium, 4.5% phosphorus, 40.0% salt (NaCl), 6.0% magnesium, 120 mg/kg cobalt, 4 000 mg/kg manganese, 250 mg/kg iodine, 200 mg/kg Se, 5 000 mg/kg zinc, 250 000 I.U./lb vitamin A, 40 000 I.U./lb vitamin D, and 200 I.U./lb vitamin E. The mineral supplement was intended to be diluted with salt to provide 90 mg/kg Se as per the FDA-regulations for free-choice minerals. Average consumption of the mineral mixture by the sheep in this trial was not determined. All sheep had equal access to the mineral product. However, as is typical of free-choice mineral products, there is significant individual variation in intake. Based on the mineral's salt content, expected daily intake would be between 5 and 10 g/head resulting in Se intake of 1-2 mg/d, which is higher than the 0.7 mg/d (0.7 mg/d is considered equivalent to 0.3 mg/kg supplemental Se in the total diet) allowed by the FDA. The FR control program in this flock consisted of a walk-through formalin foot bath (used monthly or less often). A FR-vaccination program was not used. No antibiotics for FR treatment were administered. This program continued for all sheep for the duration of the study.

### Delayed-type hypersensitivity (DTH) skin test with keyhole limpet hemocyanin (KLH)

Adaptive immune function was evaluated after 3 months of Se supplementation by immunizing all sheep twice 2-weeks apart with 0.5 mL of KLH (500 μg of KLH emulsified in 1.0 mg of T1501 adjuvant for a total volume of 0.5 mL; administered intramuscularly) as previously described [14]. One day after the second immunization, intradermal skin testing was performed. Swelling and induration following an intradermal challenge were measured in the DTH test to assess CMI response by T cells. Intradermal injections of KLH were administered in 2 wool-free sites on the ventro-lateral abdomen, and circled using a felt-tip marker, and on the ear tip (in only 40 of the 57 ewes because we had insufficient KLH). No chemical restraint was used. Separate intradermal injections of histamine and saline were administered on the ventro-lateral abdomen. Individual disposable tuberculin syringes were filled with heat-aggregated KLH; histamine base (0.1 g/L; Histatrol, Center Laboratories, Port Washington, NY, USA), a positive control; or saline (0.9%), a negative control. A 25-gauge needle was used to inject 0.05 mL of each of these intradermally. The 0.05-mL dose of heat-aggregated KLH consisted of approximately 3 mg of KLH, and was prepared as described previously [14]. Measurements of wheal diameter and thickness (ear tip) were made at 0.5, 24, 48, 72, and 96 h after intradermal injections. The test was administered by the same person to all sheep. Reactions were recorded according to the diameter of induration and erythema. A reaction larger than the negative control was considered positive. If a positive reaction to the saline control was observed, its diameter was subtracted from the other reactions. After 24 h, no reactions to the saline control were noted.

Innate immune function was evaluated 30 min after injection by measuring skin responses on the ventro-lateral abdomen to intradermal injections of histamine, KLH, or saline. Histamine produced an induration typically larger than the saline control, after which the reaction subsided. Thus, these controls served also as tests of acute inflammation, in that any reaction larger than the saline reaction, which represented the volume of substance injected, was due to an acute inflammatory reaction.

### KLH antibody titer

The KLH antibody titer was also used to assess adaptive immune function. The humoral immune response was evaluated after 3 months of Se supplementation by measuring the KLH antibody titer prior to the first 0.5 mL KLH immunization and 2 and 4 weeks after the second KLH immunization. Serum was assayed for KLH antibody titer by an indirect ELISA. Briefly, 96-well microtiter plates (Costar, Cambridge, MA, USA) were coated with 5 μg/mL of KLH (100 μL/well; Sigma Chemical Co., St. Louis, MO, USA) in a 0.1 M sodium bicarbonate buffer, sealed to prevent evaporation, and incubated at 4°C overnight. The next day the coating solution was removed and 100 μL of StabilCoat (SurModics Inc., Eden Praire, MN, USA) was added to each well to block nonspecific binding sites and plates were incubated for 30 min at room temperature. After incubation, the StabilCoat was removed; plates were re-sealed and stored at 4 °C until used. Serum samples were serially diluted (1:100 to 1:1,024,000) in 0.05 M PBS with 0.05% Tween-20 (T-PBS; pH 8.0). Each dilution was added to the plate in duplicate, and incubated for 30 min at room temperature. Positive and negative control serums were included on each plate. After incubation, plates were washed eight times with T-PBS and then 100 μL of horseradish peroxidase conjugated to rec-Protein G (Zymed Laboratories Inc., San Francisco, CA, USA) was added to each well at a previously determined dilution (1:20,000). Rec-Protein G binds to IgG immunoglobulin through their Fc regions and was used instead of an anti-species conjugate as it resulted in equivalent results with a stronger signal and less background. After another 30 min incubation at room temperature, plates were again washed with T-PBS and 100 μL of 1.6 μM ABTS/H_2_O_2 _(2,2'-Azino-bis [3-ethylbenzthiazoline-6-sulfonic acid]; Sigma) in 0.1 M citrate buffer (pH 4.0) was added to all wells. Plates were incubated at room temperature in the dark and were read when a positive reference sample reached an OD of 1.0. The OD of each well at 405 nm was determined using a Bio-Tek EL312 microplate reader (Bio-Tek Instruments Inc., Winooski, VT, USA). The antibody titer was expressed as an endpoint titer for each sample, which was calculated from a regression line of OD against sample dilution, with a threshold of 0.200 OD (approximately 3 times the OD generated by the background), using a software program (KinetiCalc, Bio-Tek Instruments Inc., Winooski, VT, USA).

### Neutrophil bacterial killing of Lactococcus lactis

Bacterial killing by neutrophils was assessed after 15 months of Se supplementation. Neutrophils were isolated using a Percoll gradient technique [15], resuspended in Hank's balanced saline solution (HBSS) plus 0.5% FBS, and counted using a Coulter counter. Briefly, 10 mL of heparinized blood was collected via jugular puncture, transported on ice to the lab, transferred into 50 mL tubes, and centrifuged at 1000 × *g *for 20 min at 4 °C in a swinging bucket centrifuge to separate plasma and buffy coats from the red cell pack. Plasma, buffy coat and one-third of the red cell pack from each tube were aspirated aseptically and discarded. The remaining red cell packs containing neutrophils were mixed with 34 mL of ice-cold PBS. Samples were layered onto 10 mL of 1.084 g/mL Percoll (Sigma Chemical Company, St. Louis, MO, USA), then centrifuged at 400 × *g *for 40 min at 22 °C. After centrifugation, the red blood cells (RBC) and neutrophils pelleted at the bottom of the tube and the mononuclear cell band remained at the sample/medium interface. Supernatant, mononuclear cell layer and Percoll were aspirated and discarded. Red blood cells were lysed using 24 mL ice-cold hypotonic lysing solution (10.56 mM Na_2_HPO_4_, 2.67 mM NaH_2_PO_4_, pH 7.3) for 90 s. Isotonicity was restored by adding 12 mL ice-cold hypertonic restoring solution (10.56 mM Na_2_HPO_4_, 2.67 mM NaH_2_PO_4_, 0.43 M NaCl, pH 7.3). Remaining leukocytes were pelleted by centrifugation at 800 × *g *for 5 min at 4 °C. A 20 μL aliquot of the cell suspension was used to determine cell concentration (Coulter ZB1 Counter, Coulter Electronics Inc., Hialeah, FL, USA). Another 5 μL aliquot was used to assess purity of neutrophil preparations (differential cell count) by microscopic examination after Wright-Giemsa staining (95 ± 1%; mean ± SEM).

Neutrophils (2 × 10^5 ^cells/well) were seeded into triplicate wells of a 96-well tissue culture plate, previously coated with poly-D-lysine. Neutrophils were activated for 10 min with 25 nM PMA (diluted in DMSO; < 1% final solution). Logarithmic phase *Lactococcus lactis *(MG1363) bacteria containing an erythromycin-resistant plasmid were grown in M17 + glucose (M17G) + erythromycin media and diluted to the desired concentration in RPMI media + 2% heat-inactivated sheep serum (Biomeda, Burlingame, CA, USA). Bacteria were added to the neutrophils at a multiplicity of infection (MOI) of 0.01. After 1 h incubation at 37 °C in 5% CO_2_, media in the wells containing neutrophils and bacteria was serially diluted and plated onto M17G medium containing erythromycin for overnight incubation and enumeration of colony forming units (CFU). Internal control wells without neutrophils were used to determine baseline bacterial counts at the assay endpoints. Percent survival of bacteria was calculated as [(CFU/mL experimental well)/(CFU/mL control well)] × 100%.

### Relative abundance of selected mRNAs specific for neutrophil migration (L-selectin, IL-8R)

Gene expression by neutrophils was evaluated after 15 months of Se supplementation. Neutrophils were isolated as described above and frozen at -80 °C. Total RNA was extracted following manufacturer's instructions using a RNeasy^® ^Mini Kit (Qiagen Sciences; Germantown, MD, USA), quantified using the ND-1000 NanoDrop Spectrophotometer (Thermo Fisher Scientific; Waltham, MA, USA), and stored at -80 °C. Aliquots of 500 ng RNA in 10 μL were used for RT-qPCR. Relative mRNA abundance were determined for L-selectin and IL-8R to evaluate gene expression of markers involved in neutrophil migration, adherence, and activation using the Taqman^® ^One-Step RT-PCR Master kit (Applied Biosystems, Branchburg, NJ, USA). Levels of ribosomal protein large subunit family member (RPL-19) mRNA were measured to normalize selected mRNA. Results were expressed as fold change using the relative quantification method of Pfaffl [16]. The abundance of target genes, normalized to RPL-19 (as the internal control) and relative to the control group of sheep, were illustrated by 2^-ΔΔCt^, where Ct was the cycle number at which the fluorescence signal of the product crossed an arbitrary threshold set with exponential phase of the PCR and ΔΔCt = (Ct_target gene unknown sample _- Ct_RPL-19 unknown sample_) - (Ct_target gene calibrator sample _- Ct_RPL-19 calibrator sample_).

The RT-qPCR oligonucleotide primers for these genes of interest are proprietary (OmniGen Research LLC, Canby, OR, USA) and were designed based on bovine DNA sequence data. To verify that these primers were amplifying sheep cDNA, RT-qPCR primers were used to amplify sheep genomic DNA. The products were run on a 2% agarose gel and the approximately 125 base-pair band was excised. The DNA fragment size was confirmed using a 50 base pair ladder, and selected bands were excised and purified from the agarose gel using a QIAquick Gel Extraction Kit (QIAGEN Inc., Germantown, MD, USA) according to manufacturer's instructions, and prepared for sequencing. Purified products were sequenced at the Oregon State University Center for Genome Research and Biocomputing (CORE Lab) using a DNA sequencer machine. Sequence analysis was performed using commercial software. The sequence obtained was used in a BLAST (National Center for Biotechnology Information, U.S. National Library of Medicine, Bethesda, MD, USA) search of all known mammalian genomic sequences. The search showed 96% sequence identity to bovine IL-8R alpha and 99% sequence identity to bovine SELL (aliases: CD62 antigen-like family member L; L-selectin; LAM-1; LECAM1; leukocyte adhesion molecule 1; leukocyte-endothelial cell adhesion molecule 1; lymph node homing receptor; lymphocyte adhesion molecule 1; selectin L), the most closely related species.

### Statistical analysis

Statistical analyses were performed using SAS, version 9.2 (SAS, Inc., Cary, NC, USA) software. Antibody titers for KLH were normalized by log_2 _transformation and are shown as such. Relative mRNA abundance were analyzed using the internal standard-adjusted Ct-values (Ct_target gene unknown sample _- Ct_RPL-19 unknown sample_). Indicators of innate and adaptive immunity were analyzed using single (PROC GLM) and repeated measure analysis in time (PROC MIXED), respectively. Selenium treatment (healthy, FR-Se, FR-Sal) or Se-status at the time of the immune assays (healthy, FR-high Se, FR-low Se) were the covariates. Ages and production stage of ewes were not available for individual ewes in the commercial flock and thus, were not included in the model. Ewes were stratified by Se status because WB-Se concentrations of FR-Se and FR-Sal sheep overlapped (Table 1) [12]. The cut-off point for high and low Se was 250 ng/mL Se in WB and was based on the median WB-Se concentration of FR-affected sheep at the time of the immune assays (3 and 15 months; [12]). For immune assays with multiple samples in time, time of sample collection and the interaction between time of sample collection and Se treatment or status were added as fixed effects. A completely unrestricted variance-covariance structure was used to account for repeated measures taken from individual ewes across time. To obtain the correct degrees of freedom, the KENWARDROGER option was invoked. The KENWARDROGER option consists of the Satterthwaite adjustment for degrees of freedom with a Kenward-Roger adjustment on standard errors, which can be used for repeated measures studies.

**Table 1:** Whole-blood selenium concentrations in healthy and footrot (FR) affected sheep treated with selenium (FR-Se) or saline (FR-Sal)*

	Median whole-blood Se (range); ng/mL		
**Month of Treatment**	**Healthy**	**FR-Se**	**FR-Sal**

0	250 (187 351)^a^	199 (109 278)^b^	202 (111 323)^b^
3	245 (145 340)^a^	261 (189 317)^a^	199 (110 295)^b^
6	274 (180 396)^b^	302 (267 403)^a^	268 (176 408)^b^
15	230 (165 302)	288 (157 416)	237 (104 344)

*Adapted from Hall et al. [12]. Footrot-affected sheep (*n *= 38) were alternately randomized into two treatment groups and at 1-month intervals injected subcutaneously with 5 mg of Se (FR-Se) or with 1-mL of saline solution (FR-Sal) for 15 months. A control group of 19 healthy sheep without FR received no injections. There were 19 sheep/group at 0 and 3 months, 18 to 19 sheep/group at 6 months, and 9 to 14 sheep/group at 15 months.

^a, b^Medians with different superscripts within month are different (*P *< 0.05).

The effect of FR on indicators of immune function was evaluated by comparing the estimated values of the healthy group with those of the FR-affected groups. The effect of Se supplementation on indicators of immune function in FR-affected sheep was evaluated by comparing the estimated values of the FR-Se with those of the FR-Sal group. The effect of Se status on indicators of immune function in FR-affected sheep was evaluated by comparing the estimated values of the FR-high Se with those of the FR-low Se group. Data are reported as least square means ± SEM. Statistical significance was declared at *P *≤ 0.05 and tendencies at 0.05 <*P *≤ 0.15.

## Results

### Foot Lesions

**Figure 1:**
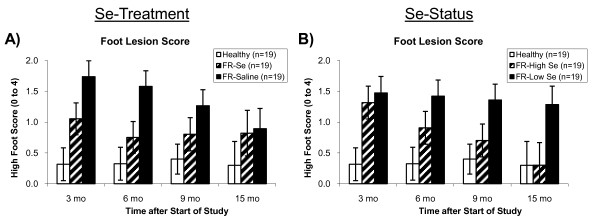
**Foot lesion scores in healthy and footrot-affected sheep treated with selenium or saline**. Footrot-affected (FR) sheep receiving monthly Se injections, had A) lower foot lesion scores (overall: *P *= 0.04), and in particular at 3 (*P *= 0.07) and 6 (*P *= 0.03) months after the start of the study. B) After stratifying FR-affected sheep by whole-blood Se (WB-Se) status at 3 months after the start of the study (WB-Se cut-off: 250 ng/mL), sheep with WB-Se concentrations above 250 ng/mL (FR-High Se) had decreased foot lesion scores by the end of the study (*P *= 0.04), whereas foot lesion scores did not change (*P *= 0.68) in sheep with WB-Se concentrations below 250 ng/mL (FR-Low Se). As a result, foot lesion scores were lower in FR-High Se sheep compared with FR-Low Se sheep (overall *P *= 0.02).

Treatment with monthly Se injections accelerated recovery from FR as previously reported [12]. Footrot-affected ewes receiving Se had lower foot scores than FR-affected ewes receiving saline (overall *P *= 0.04), with the primary differences being observed at 3 (*P *= 0.07) and 6 (*P *= 0.03) months after starting Se supplementation (Figure 1a).

To assess the effect of WB-Se status on subsequent foot scores, FR-affected ewes were stratified by WB-Se concentration (cutoff value: 250 ng/mL) at 3 months, prior to receiving the fourth monthly Se injection. Footrot-affected ewes with WB-Se concentrations of at least 250 ng/mL (FR-High Se) had lower foot scores (overall *P *= 0.02) than FR-affected sheep with WB-Se concentrations below 250 ng/mL (FR-Low Se; Figure 1b). Greater WB-Se concentrations after 3 months of Se treatment predicted future recovery from FR. Foot scores were decreased at 15 months compared to 3 months after starting the study in FR-High Se ewes (*P *= 0.04) and were similar to control sheep, whereas foot scores did not change (*P *= 0.68) and remained high in FR-Low Se ewes (Figure 1b). As a result, the primary differences for ewes stratified by WB-Se status were observed 9 (*P *= 0.08) and 15 (*P *= 0.05) months after starting Se treatment.

### Cell-mediated immunity as measured by the delayed-type hypersensitivity skin test with KLH

**Figure 2:**
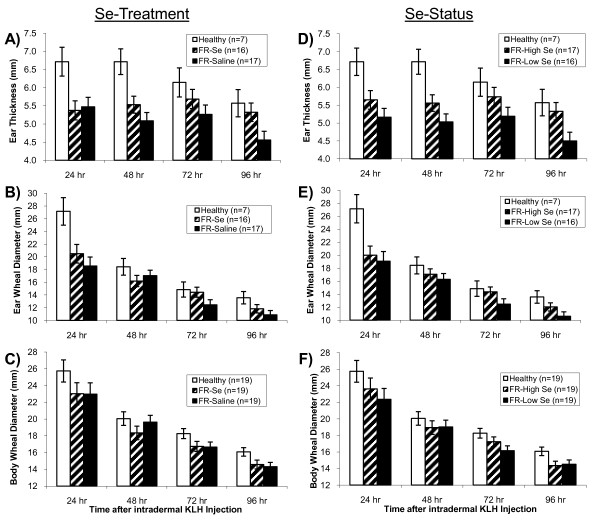
**Delayed-type hypersensitivity skin test responses in healthy and footrot-affected sheep treated with selenium or saline**. Footrot-affected (FR) sheep had an attenuated delayed-type hypersensitivity (DTH) response to keyhole limpet hemocyanin (KLH) compared with healthy sheep, as measured by A) ear thickness (overall: *P *= 0.006), B) ear wheal diameter (overall: *P *= 0.02), and C) body wheel diameter (overall: *P *= 0.04). Treatment of FR affected sheep with selenium (FR-Se) for 3 months improved the DTH response in FR-Se affected sheep, as measured by A) ear thickness (96 h after KLH challenge: *P *= 0.04 compared to FR-Saline sheep), and B) ear wheal diameter (72 h after KLH challenge: *P *= 0.09). After stratifying FR-affected sheep by whole-blood Se (WB-Se) status at the time of the immune assay (WB-Se cut-off: 250 ng/mL), sheep with WB-Se concentrations above 250 ng/mL (FR-High Se) had stronger DTH responses to KLH than sheep with WB-Se concentrations below 250 ng/mL (FR-Low Se), as measured by D) ear thickness (overall: *P *= 0.04), E) ear wheal diameter (72 h after KLH challenge: *P *= 0.10), but not by F) body wheel diameter (overall: *P *= 0.58).

Sheep affected with FR had less of a DTH response than healthy sheep, as measured by ear thickness (overall *P *= 0.006; Figure 2a), ear wheal diameter (overall *P *= 0.02; Figure 2b), and body wheal diameter (overall *P *= 0.04; Figure 2c). The location of the test site was important for assessing the duration of the DTH response; the DTH response decreased quickest for ear wheal diameter (Figure 2b). Monthly Se injections improved part of the DTH response in FR-affected sheep. Whereas ear thickness decreased from 24 to 96 h in healthy sheep and FR-Sal sheep, no significant change in ear thickness was observed in FR-Se treated sheep (Figure 2a). As a result, FR-Se sheep had a greater ear thickness than FR-Sal sheep at 96 h after KLH challenge (*P *= 0.04) and a similar ear thickness compared with healthy sheep (Figure 2a).

Footrot-affected sheep with higher WB-Se concentrations (≥ 250 ng/mL) had more intense DTH responses than FR-affected sheep with lower WB-Se concentrations (< 250 ng/mL) as measured by ear thickness (*P *= 0.04; Figure 2d). Ear wheal diameter tended to be greater in FR-High Se sheep compared to FR-Low Se sheep after 72 h (*P *= 0.10) and 96 h (*P *= 0.13; Figure 2e), whereas Se status only numerically improved the DTH response for body wheal diameter (Figure 2f).

### Humoral immunity as measured by the KLH antibody titer

**Figure 3:**
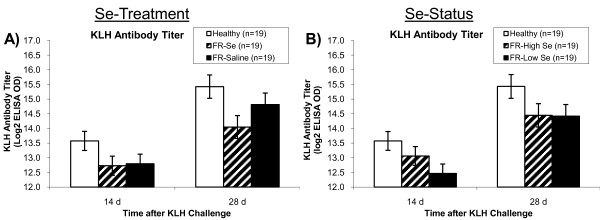
**Antibody titers to keyhole limpet hemocyanin in healthy and footrot-affected sheep treated with selenium or saline**. Footrot-affected (FR) sheep, regardless of Se treatment, had A) lower keyhole limpet hemocyanin (KLH) antibody titers 2 and 4 weeks after the second KLH immunization compared with healthy sheep (overall: *P *= 0.02). B) After stratifying FR-affected sheep by whole-blood Se (WB-Se) status at the time of the immune assay (WB-Se cut-off: 250 ng/mL), sheep with WB-Se concentrations below 250 ng/mL (FR-Low Se) had lower KLH antibody titers compared with healthy sheep (overall: *P *= 0.02), whereas sheep with Se concentrations above 250 ng/mL (FR-High Se) had intermediate values (overall: *P *= 0.09 versus healthy sheep; overall: *P *= 0.48 versus FR-Low Se sheep).

Sheep affected with FR had lower KLH antibody titers 2 and 4 weeks after KLH immunization compared with healthy sheep (overall *P *= 0.02), which was not altered by Se treatment (Figure 3a). Selenium status tended to be associated with a greater KLH antibody titer (Figure 3b). After stratification by Se status, FR-Low Se sheep had lower KLH antibody titers compared with healthy control sheep (*P *= 0.02), whereas FR-High Se sheep had intermediary values (*P *= 0.09 versus healthy sheep and *P *= 0.48 versus FR-Low Se sheep; Figure 3b).

### Innate immunity measurements

**Figure 4:**
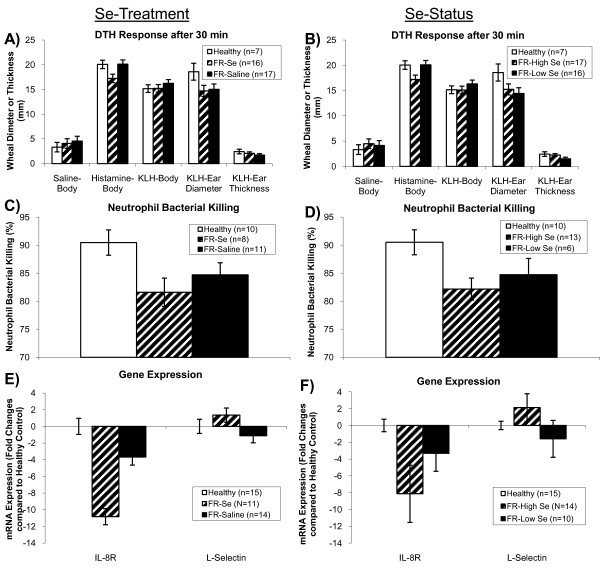
**Innate immunity measurements in healthy and footrot-affected sheep treated with selenium or saline**. Footrot-affected (FR) sheep, regardless of Se treatment, had A) smaller ear wheal diameters compared with healthy sheep 30 min after KLH challenge (*P *= 0.05), whereas Se treatment decreased the 30 min response to histamine (*P *= 0.02). B) After stratifying FR-affected sheep by whole-blood Se (WB-Se) status at the time of the DTH assay (WB-Se cut-off: 250 ng/mL), sheep with WB-Se concentrations above 250 ng/mL (FR-High Se) had a decreased response to histamine at 30 min (*P *= 0.02). C) FR-affected sheep had a lower percent neutrophil bacterial killing of *Lactococcus lactis *compared with healthy sheep (*P *= 0.04), which was not altered by Se treatment (*P *= 0.36), or D) Se status (*P *= 0.26). E) FR-affected sheep had lower mRNA concentrations of IL-8R compared with healthy sheep (*P *= 0.02), which were not altered by Se treatment (*P *= 0.24), or F) Se status (*P *= 0.32). FR-High Se status sheep tended to have increased mRNA concentrations of L-selectin (*P *= 0.07).

Sheep affected with FR, regardless of Se treatment had smaller ear wheal diameters compared with healthy sheep 30 min after KLH challenge (*P *= 0.05; Figure 4a). Monthly Se injections decreased the 30 min response to histamine (*P *= 0.02; Figure 4a). Sheep with FR-High Se status at the time of the DTH assay had a decreased response to histamine at 30 min compared with FR-Low Se sheep (*P *= 0.02; Figure 4b).

Sheep affected with FR had a lower percent neutrophil bacterial killing of *Lactococcus lactis *compared with healthy sheep (*P *= 0.04), which was not altered by Se treatment (*P *= 0.36; Figure 4c). Whole blood-Se status was not associated with altered neutrophil bacterial killing ability (*P *= 0.26; Figure 4d).

Sheep affected with FR had lower mRNA concentrations of IL-8R compared with healthy sheep (*P *= 0.02), which were not altered by Se treatment (*P *= 0.24; Figure 4e). Relative abundance of L-selectin mRNA concentrations were not affected by FR-status (*P *= 0.88) or Se treatment (*P *= 0.56; Figure 4e). Whole blood-Se status in sheep with FR was not associated with altered mRNA concentrations of IL-8R (*P *= 0.32), but tended to be associated with increased mRNA concentrations of L-selectin in FR-High Se sheep (*P *= 0.07; Figure 4f).

## Discussion

We reported previously that sheep affected with FR have lower WB-Se concentrations and that parenteral Se-supplementation in conjunction with routine control practices accelerates recovery from FR [12]. To determine whether Se acts as an immunonutrient and improves immune function in FR-affected sheep, we examined the effect of FR, Se treatment, and WB-Se status on measures of CMI, humoral immunity, and innate immunity. Our primary finding is that immune responses to a novel protein (KLH) are attenuated in FR-affected sheep with lower WB-Se status. Furthermore, the DTH and antibody titer responses to a novel protein were improved, in part, by Se treatment and high WB-Se status, supporting our hypothesis that Se acts as an immunonutrient in FR-affected sheep. Neutrophil function was suppressed by FR, but was not changed by Se supplementation or WB-Se status.

The purpose of this follow-up study was to investigate the mechanisms by which Se facilitates recovery from FR. The immune system has two functional divisions: innate and adaptive immunity. Both divisions involve various blood-borne factors (e.g., complement, antibodies, and cytokines) and cells (e.g., neutrophils, lymphocytes, and macrophages). There is large individual variation in immune function even among healthy animals. For example, differences in genetics, age, gender, levels of exercise, diet, stress, infectious disease history, vaccination status, and early life experiences are important contributors to this observed variation [17]. Thus, demonstrating an improvement in immune function with Se supplementation is challenging.

Tests used to assess adaptive immunity include the DTH test, which is also known as a type IV hypersensitivity reaction. This test provides a general measure of CMI. Professional antigen presenting cells, e.g., dendritic cells, present antigen to T lymphocytes. This results in antigen-specific activation of T lymphocytes in local tissues. Inflammatory cytokines produced by these stimulated T lymphocytes cause other mononuclear cells (lymphocytes and macrophages) to migrate to the area and proliferate. To perform this test, foreign antigen is injected under the epidermis of the skin. The immune system responds to this antigen by producing a small raised wheal that can be measured 24 to 96 h after injection. The larger the wheal, the greater is the CMI response.

In our study, healthy control sheep demonstrated an enhanced CMI response to a novel protein (KLH) compared with FR-affected sheep based on the DTH test. Results were more definitive for the ear tip compared with two wool-free sites on the ventro-lateral abdomen, likely because higher tissue compliance allowed a more diffuse DTH reaction on the abdomen. For this reason, the latter may not be as useful of a test location as the ear tip for this assay in sheep. Both ear thickness and ear wheal diameter were similarly affected, although the DTH response resolved faster over time for ear wheal diameter. The DTH response in healthy sheep receded from 24 to 96 h. Although ear thickness was suppressed at 24 h in FR ewes compared to healthy controls, there was no change across time in FR-Se treated sheep. Thus, by 96 h FR-Se sheep had a greater ear thickness than FR-Sal sheep and a similar ear thickness compared with healthy control sheep. Similarly, FR-affected sheep with higher WB-Se concentrations had a more intense DTH response than FR-affected sheep with lower WB-Se concentrations. Our results suggest an attenuated T-lymphocyte response in FR-affected sheep, which could be the result of decreased activation, migration, proliferation, or a combination of these, and which may be improved, in part, by Se treatment. An enhanced DTH response after Se supplementation was also shown by Lacetera et al. [18]. In their study, ewes given a single 5 mg Se injection 30 d prior to lambing had a greater DTH response to intradermal phytohaemagglutinin (PHA) injection at 6 h than ewes not treated with Se. Lambs born to Se-treated ewes had a greater DTH response to PHA 24 h after injection. In this study as well as ours, Se was supplemented to Se-adequate sheep. Methodology differences with injection site (ear vs. neck), antigen (KLH vs. PHA), response time (days vs. hours), and measurement technique (wheal reaction vs. skin thickness) likely account for differences between the studies.

Measuring an antibody titer in response to sensitization/immunization is another test to assess the adaptive (humoral) immune response. The animal is injected with a novel protein (e.g., KLH) that elicits an immune response. Following sensitization, antibody titers to KLH can be measured. Our results showed that FR disease was associated with lower KLH antibody titers 2 and 4 weeks after KLH immunization compared with healthy sheep, suggesting a lower antibody titer to a novel protein in FR-affected sheep. Stratification by WB-Se status showed that sheep with FR-High Se had KLH antibody titers more similar to healthy control sheep 14 days after the second immunization. There was no difference in antibody titers between FR-Se and FR-Sal treated ewes at 28 days, suggesting that titers may rise more rapidly in FR-Se treated ewes, similar to what we have shown in cattle [19]. Our results suggest that Se may improve antibody production in response to a novel protein in FR-affected sheep. It would be interesting to look at earlier titers in future studies, for example at 7 days after the second immunization.

Sheep affected with FR, regardless of Se treatment had smaller ear wheal diameters compared with healthy sheep 30 min after KLH challenge. This is consistent with a reduction in the type I hypersensitivity reaction normally induced by histamine and inflammatory cytokines. The KLH stimulates inflammatory cytokine production. Bacterial infection may cause immunosuppression of Type I hypersensitivity by affecting the release of histamine, or virulence factors such as leukotoxin, endotoxin, haemolysin, haemagglutinin and adhesin that are used to overcome the host's defense mechanisms when infection is established may also suppresses the immune response.

Selenium treatment decreased the 30 min skin-test response to histamine in FR-affected sheep. Histamine normally increases capillary permeability and relaxes vascular smooth muscle, allowing edema fluid accumulation. Influx of proinflammatory cytokines triggers production of reactive oxygen species (ROS). When produced in excess, ROS are important mediators of cell and tissue injury. Because Se is involved in redox reactions, and immune activation is usually associated with increased production of ROS by cells of the immune system, higher Se may help suppress tissue damage caused by ROS (reviewed in Murr et al. [20]). As a component of the glutathione peroxidase family of enzymes, Se contributes to the reduction of hydroperoxides in cells. Glutathione peroxidase reduces ROS to less reactive metabolites, decreasing oxidant stress. We suggest that Se suppressed oxidative stress and attenuated the early skin-test reaction at 30 min. Thus, Se may protect from type I hypersensitivity reactions, involving IgE antibody triggering of mast cells and oxidative insult, by preventing lipid hydroperoxide accumulation. On the other hand, Se enhanced the type IV hypersensitivity reaction at 96 h.

Neutrophils are the most numerous and important cellular component of innate immunity. Their primary functions are phagocytosis and destruction of microorganisms. They serve as the body's first line of defense against invading microorganisms. Phagocytosed bacteria are rapidly killed by proteolytic enzymes (e.g., myeloperoxidase), antimicrobial proteins, and ROS when membrane-bound granules fuse with phagocytic vesicles. In addition to phagocytosis, bacterial killing occurs via neutrophil extracellular traps (NETs) [21-23]. Upon activation, neutrophils release extracellular fibers called NETS, which form a meshwork that kills bacteria extracellularly without the need for phagocytosis. NETs are made up of DNA, histones, and granule proteins such as elastase. Both Gram-positive and Gram-negative bacteria are killed, which suggests that NETs can kill a wide range of pathogens. These NETs serve as a physical barrier, which prevents the spread of bacteria.

To assess innate immunity of neutrophils, a biologic assay was performed using *Lactococcus lactis *and measuring percent bacterial killing. Neutrophils from healthy control sheep demonstrated higher percent bacterial killing compared with neutrophils from FR-affected sheep, regardless of 15 months of Se supplementation. Even when FR-affected sheep were stratified by WB-Se status, neutrophil bacterial killing ability was not altered. Our results suggest that FR-affected sheep have reduced neutrophil bacterial killing ability, which cannot be improved by Se supplementation or high WB-Se status. It has been reported previously that neutrophils of Se-deficient cattle have reduced ability to kill phagocytosed bacteria [24,25]. Although acute infections have been shown to decrease serum-Se levels [26], sheep in the current study were Se-adequate. It is possible that supranutritional Se does not further improve neutrophil bacterial killing ability or that the disease state inhibits or prevents modulation of neutrophil bacterial killing ability.

Measuring the relative abundance of mRNA specific for neutrophil migration activity (L-selectin and IL-8R) by RT-qPCR is another method for assessing innate immunity. To protect against invading pathogens, neutrophils migrate to infected sites. Neutrophils roll along walls of blood vessels by the coordinated formation and breakage of selectin-carbohydrate chemical bonds. L-selectin is a cell surface glycoprotein that is constitutively expressed on the surface of most leucocytes [27]. L-selectin is important for the binding and subsequent rolling of leucocytes along endothelial walls, facilitating migration into secondary lymphoid organs (e.g., naive T cells) and into sites of inflammation (e.g., neutrophils) [27]. Chemokines such as IL-8 and its receptor control the interaction of neutrophils with the epithelial cell barrier. IL-8 is recognized mainly for its ability to induce neutrophil migration, but it also increases cytokine production, enhances phagocytosis and ROS generation, and regulates cell survival [28-30]. In our study, we found that sheep affected with FR had lower concentrations of IL-8R mRNA compared with healthy sheep, and Se treatment had no effect. Our results suggest that attenuated IL-8R gene expression may play a role in reducing bacterial killing ability in FR-affected sheep; IL-8R gene expression, however, may not be responsive to Se treatment. The relative abundance of L-selectin mRNA was not affected by FR-status, however, mRNA concentrations tended to be higher in FR-High Se sheep, suggesting that L-selectin is a potential molecular target of Se treatment.

In conclusion, our goal was to determine if supplementing Se at concentrations above those currently recommended for sheep (supranutritional) can modulate the immune response in a way that reduces severity and/or improves recovery from a disease process. We reported previously that parenteral Se supplementation in conjunction with routine control practices resulted in higher WB-Se concentrations and more rapid improvement of foot lesions [12]. In this study, we found that neutrophil function and the DTH and antibody titer responses to a novel protein (KLH) were attenuated in FR-affected sheep. The DTH and antibody titer responses to a novel protein were improved, in part, by Se treatment and WB-Se status, suggesting that FR in sheep is associated with reduced lymphocyte function, which may be partly restored by improving WB-Se status (≥ 250 ng/mL). Additional experiments are needed to determine the best source, route of administration, and dose of Se to optimize immune function in sheep with FR.

## Abbreviations

CFU: colony forming units; CMI: cell-mediated immunity; DTH: delayed-type hypersensitivity; FR: foot rot; FR-Se: foot-rot ewes receiving selenium injections; FR-Sal: foot-rot ewes receiving saline injections; KLH: keyhole limpet hemocyanin; Se: selenium; T-PBS: Tween-PBS; ABTS: 2,2'-Azino-bis (3-ethylbenzthiazoline-6-sulfonic acid); HBSS: Hank's balanced saline solution; MOI: multiplicity of infection; PHA: phytohaemagglutinin; RBC: red blood cells; ROS: reactive oxygen species; RPL-19: ribosomal protein large subunit family member-19; WB: whole blood.

## Competing interests

This manuscript, in whole or in part, has not been published elsewhere, and has been read and approved by all authors. Sources of extra institutional funding or support have been acknowledged. There are no financial or personal conflicts of interest. The authors represent and warrant that our part of the work as submitted will in no way violate any copyright, or any other right.

## Authors' contributions

JAH conceived the study, participated in its design, coordination, and field work, and drafted the manuscript. RLS participated in the laboratory work and contributed to writing the manuscript. RMC, WRV, YW, and BVS participated in the laboratory work. DPB, KNT, NEF participated in the field work and the laboratory work. RJVS and GB participated in the design of the study and performed the statistical analysis. All authors read and approved the final version of the manuscript.
